# Litter Management Practices and House-Soiling in Italian Cats

**DOI:** 10.3390/ani13142382

**Published:** 2023-07-22

**Authors:** Alessandra Tateo, Claire Ricci-Bonot, Martina Felici, Martina Zappaterra, Leonardo Nanni Costa, Katherine Houpt, Barbara Padalino

**Affiliations:** 1 Department of Precision and Regenerative Medicine and Ionian Area (DiMePRe-J), University of Bari, 70124 Bari, Italy; alessandra.tateo@uniba.it; 2 Animal Behaviour, Cognition and Welfare Group, School of Life Sciences, University of Lincoln, Lincolnshire LN6 7TS, UK; criccibonot@lincoln.ac.uk; 3 Department of Agricultural and Food Sciences, University of Bologna, 40127 Bologna, Italy; martina.zappaterra2@unibo.it (M.Z.); leonardo.nannicosta@unibo.it (L.N.C.); barbara.padalino@unibo.it (B.P.); 4 Department of Clinical Sciences, College of Veterinary Medicine, Cornell University, New York, NY 14850-9535, USA; kah3@cornell.edu

**Keywords:** behavior, elimination, litter, management, welfare

## Abstract

**Simple Summary:**

House-soiling is one of the commonest behavioral problems in cats and one of the main reasons why cats are abandoned at shelters. This study aimed to document the litter management practices and the recalled prevalence of elimination problems in a representative sample of the Italian pet cat population. An online survey collected data for a total of 3106 cats. Cats were mostly European adult-aged and living in apartments with other pets. They were mainly provided with covered litter boxes filled with clumping substrates, and the cleaning of the litter box and its full replacement took place daily and weekly, respectively. Professionals and amateurs owned cats with characteristics oriented toward breeding and companionship, respectively. Professionals provided fewer square meters per cat to their cats but were more diligent in litter box cleaning compared to amateurs. House-soiling was reported by 16.7% of the respondents. It was mainly related to urine elimination on objects and was lower than in other investigated populations. The demographic information collected has increased our knowledge and may be useful to enhance cat management in Italy.

**Abstract:**

There are about 10.1 million domestic cats in Italy, but information on cats’ litter management and house-soiling prevalence is scant. This study described cats’ and cat owners’ profiles, litter management practices, and whether cats show house-soiling, also comparing between professionals (i.e., breeders) and amateurs (i.e., pet owners). A cross-sectional online survey sought respondents’ housing, family, and cat details, as well as other pet details, litter details, and whether the cats showed house-soiling. Data for a total of 3106 cats were obtained. Italian cats lived mainly in apartments, along with other cats or dogs. Italians owned mostly adult European breed cats, to whom they provided covered litter boxes filled with clumping substrates, scooped daily, and completely replaced weekly. Litter cleaning was more frequent when cats were owned for financial purposes (i.e., breeders) rather than for companionship, but more space was provided for pets than for breeding cats. The recalled prevalence of elimination problems (16.7%) was lower compared to other studies, with cats mainly eliminating urine (54.6%) on objects in squatting posture (35.2%). Overall, this research increased our understanding of cat litter management in Italy. These findings could fill a gap in the knowledge regarding litter management and house-soiling incidences in Italy. Further studies to investigate possible risk factors for house-soiling are needed.

## 1. Introduction

Cats are among the most popular animals worldwide [1]. In the United Kingdom (UK), in 2016, 24% of the population had a pet cat, for a total of 10.5 million pet cats throughout the country. Similarly, in Australia, there were 3.3 million pet cats, with 29% of households owning a cat, and the USA followed a similar trend, with 30.5% of households owning 74 million pet cats [1]. In Europe, in 2021, 26% of cat/dog-owning households owned a cat, for a total of 113.5 million pet cats [2]. In Italy, the situation is in line with these trends, and the population of domestic cats is estimated to have reached 10.1 million, compared to 8.7 million for dogs, with a percentage of 22% of pet-owning households owning one or more cats [3]. However, estimating the cat population in Italy is difficult since cat identification and registration are voluntary [4].

In Italy, a microchipped cat can be registered in the Feline Unit of the public regional Canine Registry or a private Feline Registry managed by the Italian Association of Veterinarians [4]. At present, in the whole country, 1,166,386 cats are recorded in the public registry (mainly composed of the feral cat colonies managed by the Local Health Unit) [5] and 86,586 cats in the private one [6]. Since most Italian cats are not officially registered in databases, information about the demographics of the cat population, the ownership profiles, and the management of these cats are scant [4]. However, this information could be valuable, both for veterinary industries and practitioners [4], who are interested in animal disease control, zoonoses risk assessment, animal welfare issues, and stray population management, both for health and economic purposes [4]. Welfare in domestic animals is often investigated less than that of livestock [1]. This is because pets live in close contact with humans, leading to the assumption that pets are in good welfare conditions [1]. However, cat management should be adapted to cat needs and factors, like a suboptimal home environment could lead the cat to the manifestation of various behavioral problems [7]. Some studies reported “house-soiling”, also named inappropriate elimination, as one of the commonest behavioral problems in cats [8].

Inappropriate elimination includes any deposition of urine (periuria) and/or feces (perichezia) outside the litter box and is one of the main behavioral reasons why cats are abandoned at shelters [9,10,11]. A stressful environment, a multi-cat or multi-dog household, incorrect litter box management, and medical problems seemed to predispose the cat to house-soiling [12]. Litter management is especially important because, even when the soiling is of medical origin, it may persist after the medical problem has been resolved [8]. In fact, in all cases of periuria and perichezia, proper litter management should always be considered a prerequisite [12]. The commonest recommendations to properly manage the litter and litter box are the following: daily litter cleaning and weekly litter full replacement; using fine-grained clumping materials as substrates at an appropriate depth (approximately 3 cm); one litter box per cat plus one; litter box size adapted to the size of the cat; and location far from water, food, and busy thoroughfares [12,13]. Therefore, litter and litter box cleaning, the size of the litter granule, the number of litter boxes, the location, and the size are all important factors to consider [14].

To properly investigate behavioral or health problems in pet cats, it is vital to first have appropriate knowledge concerning the quantitative data on the demographics of the reference population [15]. This survey aimed to better understand pet cats’ living environments and litter management and document the manifestation of elimination problems in an Italian pet cat population, comparing professionals (i.e., breeders) and amateurs (i.e., pet owners).

## 2. Materials and Methods

### 2.1. Respondents

The target population was Italian people who owned at least one domestic cat and one litter box. A power analysis [16] determined that 2737 survey responses by cat owners would be representative of the Italian cat population, which was estimated at 10.1 million in 2022 [3]. The minimal sample size was determined, assuming an expected proportion of inappropriate elimination of 65% [17,18], with 3% absolute precision and 99.9% confidence interval (CI). The expected proportion of inappropriate elimination was obtained by averaging the proportions of inappropriate elimination found during a clinical assessment of behavior by Sung and Crowell-Davis [17] and Cannas et al. [18] (i.e., 59–79% and 51.2%, respectively).

### 2.2. Survey

This cross-sectional online survey (see Table S1) was conducted In Italy from March to May 2022. The survey consisted of 18 closed and 3 open-ended questions asking for respondents’ housing details (i.e., housing type, housing size), respondents’ family details (i.e., number of adults, number of children under 7 years old, number of children between 7 and 12 years old), respondents’ pet details (i.e., number and species of owned animals, other than cats), cat’s respondent details (i.e., relationship with the cat/s, number of cats, cat gender, cat breed, cat age), litter details (i.e., number of litter boxes, type of litter box, type of litter, litter scooping frequency, litter full replacement frequency), and whether the cat showed inappropriate elimination. For the cats suffering from house-soiling, a further set of 4 questions about elimination type, locations of the eliminations, posture, and whether the cat was suffering from a medical problem were asked.

The survey was developed through a process of iterative review by the authors. The survey was built in Italian, using Qualtrics Software© (Qualtrics^XM^, Provo, UT, USA, 2023) [19], piloted through Facebook among authors’ acquaintance cat owners, and adjusted in response to feedback. Italian invitation letters and links to the survey were disseminated through social media (Facebook, Instagram, WhatsApp, and LinkedIn), associations and veterinary institutions (see Table S2).

The authors contacted Facebook pages and Instagram profiles, respectively, via chat or directly to ask them to post the invitation letter and the link on their pages/profiles. The link was also promoted on LinkedIn, being posted on the personal page of the authors. On WhatsApp, groups or individuals known to the authors were contacted, and they were asked to disseminate the survey link. Italian cat associations and Veterinary Institutions were contacted via mail, and they disseminated the questionnaire by posting it on their web pages and sending emails and newsletters with the link to their members. Survey details were shared on the internet and social media, reaching people not directly contacted by the authors, in a social media version of “snowball sampling” [20]. The survey link was available for completion between 15 March and 15 May 2022 (~2 months).

### 2.3. Data Handling and Definition of the Variables

The survey responses collected in Qualtrics were exported and organized in Microsoft Excel^®^ (Microsoft^®^ Excel^®^ for Microsoft 365 MSO (Version 2306 Build 16.0.16529.20100)) for descriptive analysis. Answers from the 45 respondents who did not have a litter box for their cat were then removed from the dataset, as they would not fit the aim of this research (i.e., a better understanding of litter management and documenting the manifestation of house-soiling).

The variables number of cats and number of litter boxes were initially considered numeric to calculate the number of litter boxes per cat (i.e., the ratio between the number of cats and the number of litter boxes). Then, all the following quantitative data were transformed into categorical variables: housing size, number of adults, number of dogs, number of cats, square meters per cat, and number of litter boxes. Where possible, categories with an insufficient number of answers (i.e., less than 5% of answers) were combined [21]. This was the case for the following categorical variables: “cat’s age”, “type of litter”, “litter scooping frequency”, and “litter full replacement frequency”. For the last three variables, as it was impossible to combine all the categories under 5% (e.g., different types of litter, “never cleans”, “cleans when needed”), a category “Other” has been added. For the breed of cats, all breeds represented less than 5% have been grouped in the category “Other”, except for “Persian” and “Siberian”.

The answers to the questions about the number of children above 7 years old or between 7 and 12 years old were transformed into a dichotomous variable (i.e., “Presence/Absence”). Similarly, the answers to the question “Are there other pets in the family?” were transformed into a dichotomous answer: “Yes/No” for the presence of dogs and the presence of animals other than cats and dogs. The length of cat’s hair (Short/Long) was determined by the breed of the cat. Information about the presence and access to a garden was extracted from respondents’ answers, combining the text written in the option “Other” of questions 1, 11, 12, and 13. Respondents specified if they were living in a house with a garden or on a farm, whether their cats had the possibility to go outdoors and eliminate outside, and whether they had some litter boxes. In the case where they had no litter boxes, their answers were not further analyzed. For the variable “Origin”, all respondents who were not in Italy when they filled in the questionnaire were put in the “Other” category. For the variable “Relationship with the cat”, professionals were the people involved with cats for financial reward (i.e., breeders), while amateurs were people who took care of cats for no financial purpose (i.e., pet owners).

Furthermore, some questions were asked only in cases where the respondents answered that their cat expressed house-soiling. Those questions were related to the type of house-soiling (“What does the cat eliminate outside the litter box?” Possible answers: “Urine”, “Feces”, or “Both”); the location where the cat eliminates (“Where does the cat eliminate?” Possible answers: “Same spot” or “Different spots”; “Where does the cat eliminate precisely?” Possible answers: “Bedroom”, “Floor”, “Near the litter”, “Objects”, “Outside the house”, or “Absorbent mat”); the posture while eliminating (“Posture when the cat eliminates outside the litter box?” Possible answers: “Squatting”, “Standing with tail raised”, or “Have not observed”); and which type of health problem does the cat have (open-ended question). The answers obtained to the question about cat’s health problems were transformed into two variables: a dichotomous variable named “Presence of cat’s health problems” (presence/absence) and a categorical variable named “Type of cat’s health problems” containing three classes: “healthy”, “urinary tract disease”, and “Others” (others contained musculoskeletal problems, digestion problems, and Feline Immunodeficiency Virus). Table S3 shows the names, definitions, and categories of all the variables considered.

### 2.4. Statistical Analysis

Descriptive statistics of all numeric, categorical, or dichotomous variables were performed using the Statulator^®^ online free software (2023) [22] and reported as counts and percentages. Chi-squared tests were conducted to determine the association between the number of cats owned and the litter scooping frequency, amateur/professional status, and all the other categorical variables in the dataset, excluding variables related to the type of elimination and elimination characteristics (i.e., the spot of the elimination, precise location of the elimination, posture when eliminating, and presence and type of health problems). Chi-squared was performed in the R environment (R Version 4.2.3) [23].

## 3. Results

### 3.1. Response and Response Rate

We received 2839 responses. Of those, 2569 respondents replied for one cat to the questionnaire (90.49%), whereas 225 (7.93%) and 45 (1.59%) respondents answered, respectively, for two and three cats to the questionnaire, for a total of 3154 cats. Unfortunately, 45/2839 (1.59%) respondents filled in the questionnaire but did not have a litter box, so their answers had to be eliminated (i.e., inclusion criteria not met). Therefore, data from only 3106/3154 cats were retained, and this could be considered a significant sample size. The completion rate was 84% since 497/3106 surveys were not fully completed, leading to missing data in some final questions.

### 3.2. Descriptive Statistics

The median number of cats owned by the respondents was 2 (IQR: 1–3; Min.–Max.: 1–30), and the median number of litter boxes provided to the cats was 2 (IQR: 1–3; Min.–Max.: 1–30). The median number of litters per cat was 1 (IQR: 0.50–1; Min.–Max.: 0.03–5), while in multi-cat household, the owner provided their cats with a median number of 0.7 litters (IQR: 0.50–1; Min.–Max.: 0.03–3.8).

The counts and percentages of the answers obtained for the survey are reported in Table 1. Most respondents who filled out the questionnaire were from Northern Italy (52.74%) and were living in an apartment (69.86%) without a garden (69.51%). Most of the households consisted of two adults (47.66%), without children (more than 90%), and without animals other than cats (69.80%). Most of the respondents were amateurs (91.85%) and possessed two or three cats (44.85%) that were older than 5 years (42.17%), neutered (84.03%), and of European type (50.64%). The cats mostly had 25 to 49 m2 per cat (32.19%), only one litter box available in the house (47.33%), which was commonly covered (51.38%), and located in the bathroom (51.92%). The most used litter was clumping (52.48%), followed by the biodegradable one (20.15%), which was scooped at least once a day (80.58%) and fully cleaned at least once a week (59.11%). House-soiling was reported for 520/3106 cats (16.74%) (Table 1).

**Figure 1:**
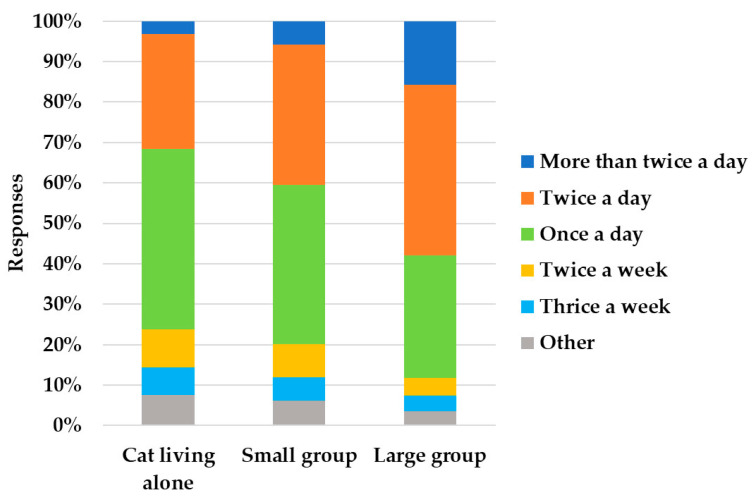
Association between the number of cats and the litter scooping frequency calculated based on the 3106 cats’ details received with an online survey in Italy.

There was an association between the number of cats owned by the respondents and the litter scooping frequency (Table S4), with an increase in litter scooping frequency as the number of cats owned increased (*X*^2^ = 176.25; *p* < 0.001) (Figure 1).

**Figure 2:**
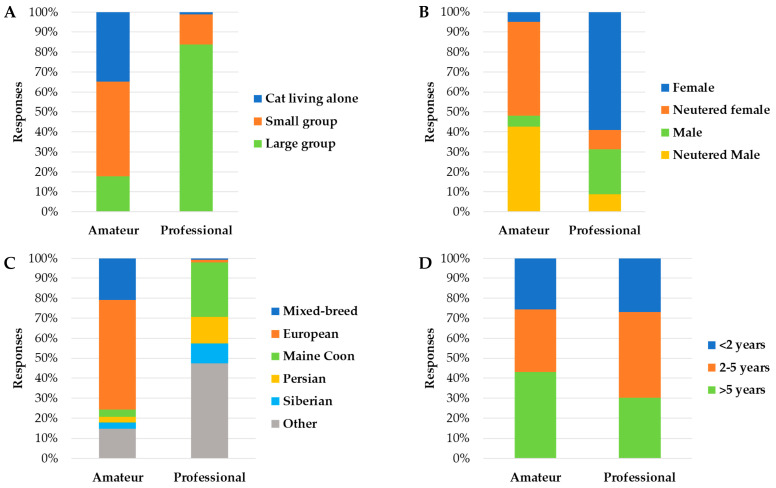
Associations between the relationship with the cat (i.e., amateur or professional) and the number of cats (**A**), cat’s gender (**B**), cat’s breed (**C**), and cat’s age (**D**), calculated based on the 3106 cats’ details received with an online survey in Italy.

**Figure 3:**
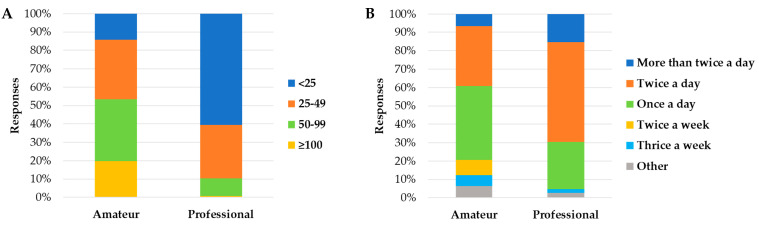
Associations between the relationship with the cat (i.e., amateur or professional) and the square meters per cat (**A**) and the litter scooping frequency (**B**), calculated based on the 3106 cats’ details received with an online survey in Italy.

There were also many significant associations between the relationship with the cats (i.e., amateur or professional) and the respondents’ details, the respondents’ housing, family and pets’ details, and the litter management (Table S5). Professionals owned more cats (*X*^2^ = 575.16; *p* < 0.001) than amateurs, and their cats were younger (*X*^2^ = 18.77; *p* < 0.001), mostly intact (*X*^2^ = 981.16; *p* < 0.001), and purebred animals (*X*^2^ = 657.83; *p* < 0.001) (Figure 2). Cats owned for breeding purposes were kept in smaller space allowances (*X*^2^ = 364.06; *p* < 0.001) than companion cats, but their litters were scooped more frequently (*X*^2^ = 101.70; *p* < 0.001) (Figure 3). There were instead no associations between the relationship with the cats and the variables named cat per respondent (*X*^2^ = 0.17; *p* = 0.920), children under 7 years old (*X*^2^ = 1.11; *p* = 0.292), animals other than cats and dogs (*X*^2^ = 0.01; *p* = 0.941), boxes located under the stairs (*X*^2^ = 0.18; *p* = 0.672) or in the garden (*X*^2^ = 0.93; *p* = 0.335), and cats eliminating outside the litter box (*X*^2^ = 0.06; *p* = 0.812).

Table 2 reports the counts and percentages for the questions related to the cats that eliminate outside the litter boxes. In the case of the 520 cats eliminating outside their litter, urinary house-soiling (54.60%) was more frequent than fecal house-soiling (24.90%) and concurrent urinary and fecal house-soiling (20.50%). Cats mainly eliminated in the same spot (64.64%), more commonly on objects (31.66%), or near the litter (28.25%). The posture during elimination was equally distributed between squatting (35.24%) and standing with the tail raised (30.57%), while in one-third of cases, the posture of elimination was not observed (34.18%). A health problem was recalled or known for only a small percentage (17.12%) of the cats (Table 2). Among the cats eliminating outside the litter boxes and having health problems, almost half were affected by urinary tract diseases (44.94%).

## 4. Discussion

The results of this survey documented the demographic and living environment characteristics of cats, owners’ litter management practices, and the recalled prevalence of house-soiling in a population of Italian domestic cats, comparing them between cat breeders and pet owners. Our findings can be useful to industries that supply feline products, to veterinarians, and to owners by adding a piece of information regarding the living conditions and the management practices related to Italian cats.

Our cats’ living conditions were in line with other studies conducted both in Italy and in other countries [3,24,25,26]. Respondents lived with their cats more frequently in apartments, as reported in the Italian annual report of the Association of Pet Food and Pet Care Enterprises [3], with 25–49 m^2^ available per cat, in line with what Heidenberger et al. [24] indicated. However, in our case, the percentage of respondents having a garden was lower than that reported in other studies [26]. This was expected since the majority of the respondents were from Northern Italy, which is highly urbanized, and people usually live in multi-level buildings containing a high number of small apartments. In the present survey, a specific question about cats’ possibility to access outdoors was not included. This was because our investigation focused mainly on litter management and house-soiling in cats. However, since the possibility to go outdoors is an important aspect of cat management, further studies should consider this aspect.

Professional cat owners tended to have bigger houses (i.e., ≥151 m^2^) compared to amateur owners. At the same time, professionals owned more cats than amateurs, providing, therefore, smaller space allowances per cat. This could be one risk factor for breeding cats since it has been reported that some commercial dog breeding establishments tend to keep their animals within the minimal space required by the law (when a law is present), and this can lead to behavioral problems in the adult dog [27]. A critical minimum space allowance has not yet been determined for good cat welfare. However, providing cats with enough space to perform their behavioral patterns, access all the environmental resources without sharing, and avoid seeing other cats and people (if they choose to do so) is known as crucial for cat welfare [28]. Therefore, regardless of the type of management (e.g., oriented toward breeding or companionship), what matters is the awareness and respect of cats’ needs and the implementation of good care practices to avoid health and welfare problems associated with each management style [29].

Within cats’ housing details, the household composition is another critical point to respect for the cat’s needs. In our study, respondents were mostly part of two-person households with no children, and most of the cats lived with other animals, especially other cats or dogs. This is in agreement with Italian statistics on the number of pets per household, which were reported to be an average of 2.16 pets per household [3]. In our case, as with other studies, cats lived in households with other cats or dogs [26,30]. Living with other animals can be a source of stress for cats. Multi-cat households, or cats and dogs in the same household, were considered by many scientific studies as risk factors for the development of behavioral problems such as aggression or house-soiling [31,32]. For house-soiling, litter box management, such as cleanliness and location, becomes critical if there are multiple animals other than an individual cat in the household [31]. Dogs and other cats can be **“**obstacles**”** for the cat to reach the litter box freely [32]. In addition, in multi-cat households, the number of litter boxes should equal the number of cats plus one additional box so that competition for resources would be limited [31]. Some studies reported that it is not the number of cats per se creating stress, but competition for common resources, especially in confined spaces [33,34]. That is why it would be recommended that breeders and owners with multiple pets pay special attention to each of them. Particularly for the cat, quiet places, hiding places, or places in the house that cannot be reached by dogs are fundamental for avoiding the “stresses” of the household and reaching all the resources needed [31]. The litter should also be located in a discrete corner, away from other resources such as food, water, and transited spots [13,35]. Unfortunately, in our study, we did not investigate if the litter boxes were located near food or water. As this could represent a critical point for the litter box’s attractiveness, this information should be asked for in future studies.

Concerning the demographic characteristics of the pet cat population investigated in the present study, the cats were mainly neutered, European-breed, and adults. The prevalence of neutered cats found in our study reflects the currently common practice of neutering pets routinely in Italy, as well as in other countries [26,36,37]. When considered in association with being professionals or amateurs, there was an expectedly different distribution among gender. Professionals owned more intact cats than amateurs since they use the cats for breeding purposes. The European-breed cats were the most represented in our study, in line with what was reported in other studies conducted in Italy [4,30]. However, not surprisingly, professionals owned mostly purebred cats compared to amateurs. Main Coons, Persians, and, in general, long-haired cat breeds were more common among professionals in Italy. This is in line with the surveys carried out in France [38] and Sweden [39]. Most respondents owned adult cats, which is in agreement with other surveys conducted in Italy [26,30]. The adult age of the cats we recorded was probably linked to most respondents living in urban areas, as suggested by the higher percentage of apartments as a housing type, where the average age of the cats seemed to be higher than in rural areas [4]. This tends to be because in urban areas, cats are kept inside and are cared for more by their owners than in rural areas [29]. The age categories of the owned cats differed between professionals and amateurs. Amateurs owned mainly mature cats (>5 years) [40], which is in line with the literature [26], suggesting the companionship purpose of these animals. Conversely, professionals owned mostly adult cats [40], aged from 2 to 5 years. This was expected since this is the most common range of age (i.e., from 18 months to 6 years) for breeding queens and toms [28], which was also reported in surveys spread among French [38] and Swedish [39] cat breeders (average age of the queen is 3.3 years).

As part of cat care and management, we asked respondents about their litter box management habits. The majority of the respondents provided one litter box per cat, but in particular in multi-cat households, the common situation was that the cats had less than one litter each. This is contrary to what scientific studies suggest [31,32]. In fact, the “obstacle” of feces or urine in a dirty litter box due to previous use seems to prevent its reuse [41]. Moreover, a strong odor because of insufficient cleaning seems to make the use of litter boxes less likely [32,42]. However, the most reported litter scooping frequencies in our survey were once/twice a day, with full litter replacement frequency occurring mainly once a week. These frequencies were in line with what was recommended for optimal litter management [12]. In addition, Grigg et al**.** [12], in their study, suggested increasing the frequency of litter scooping, especially in multi-cat households [12]. This good practice was performed by the respondents in our study, since as the number of cats increased, the litter scooping frequency increased. In our study, professionals scooped the litter more frequently than amateurs. This could still be due to the fact that professionals have more cats, so they tend to clean the litter boxes more, in line with the recommendations reported by Grigg and colleagues [12].

In addition to the number of litter boxes, their location and substrates also have an important role in respecting cats’ needs. Our survey respondents located the litter boxes, especially in the bathroom or in the bedroom. The general rule of thumb is that litter boxes should be in the core area where the cat lives, in a quiet place that is easily accessible, especially for senior cats, and without conflict over their use, especially in multi-cat households [32,43]. Regarding the litter details, most respondents reported providing their cats with covered litter boxes filled with clumping substrates. Although no clear preference for the type of litter box has been identified [12], each type can have its advantages and disadvantages. A covered litter box offers the advantages of limiting material dispersion, trapping odors, and allowing the cat to eliminate in a place protected from external stressors (i.e., children, other animals, other cats, etc...) [31,32]. However, because of trapping odors, a covered litter box managed improperly could induce avoidance and inappropriate elimination in the cat. In addition, the coverage may inhibit the owner’s ability to perceive the litter box’s level of cleanliness, and this would compromise its use by cats [31]. Lastly, a covered litter box type should also respect the size of the cat and be easily accessible [32]. Thus, a covered litter box can be a valid type of litter box; however, certain precautions should be taken, such as recognizing the characteristics of the cat for which it is provided and ensuring proper cleanliness management. The clumping litter was the type most used by our respondents, as recommended by several scientific papers [12,31,32]. Unscented, fine-grained clumping litter was considered optimal for cats [12], and in 2020, it was reported that clumping was the litter most often used by Italians [44]. However, as a second preference, our respondents reported using biodegradable litter, suggesting an increased focus on the impact that pets and their management have on the environment. In our survey, there were no questions about the depth of the litter substrate. However, since this is an item of preference for cats [13], future studies should include questions about it. Overall, the litter box management reported by the pet cat owners in our study seems to be in line with what the literature indicates as optimal, suggesting owners paid close attention to the needs of their pet cats.

Approximately 17% of cats included in our dataset exhibited house-soiling (sometimes referred to as inappropriate elimination, but it is not inappropriate for the cat), and this is lower than that reported in the literature [18,41]. In Australia, using a similar online survey, 2371/11955 (19.8%) of respondents reported that at least one of their cats showed house-soiling [43]. In Italy, 51.2% of the cats presented at vet clinics were diagnosed with house-soiling [18] among cats suffering from behavioral problems. However, while the other prevalence could be under or over-estimated, ours could be a value close to the real Italian situation. Italian cats seem to more frequently exhibit urination outside the litter box, mainly in one spot, represented by objects or spots near the litter, both squatting and standing with tail-raised positions. The deposition of urine in a squatting position is mainly attributed to the motivation to eliminate and consists mainly of a large deposit of urine on a horizontal surface [32,35], whereas the deposition of small amounts of urine on vertical objects is attributed to the motivation to mark. Usually, house-soiling is associated with various factors, such as improper litter box management, aversion toward litter box substrates, competition with other cats in the case of multi-cat households, or due to an aversion, in general, to the litter box location or to the litter box itself [8,31,32,35]. In addition, health problems, such as urinary or musculoskeletal (e.g., arthritis in senior cats) diseases, may also predispose to the manifestation of house-soiling, as already shown in the literature [8,31,37,43]. Usually, solving house-soiling depends on understanding the factors, clinically or management-related, behind it, which may be related to the characteristics of the litter box, the living environment, or the cat itself [7]. However, the identification of the factors that may have increased or decreased the occurrence of house-soiling in the studied cats would require further investigation.

Elimination should be distinguished from marking behavior [35]. Marking behavior is known as spraying, whose predisposing factors are different from those of elimination. Spraying in cats is the behavior in which the cat backs up to a vertical object and sprays urine horizontally, with the tail held straight up and usually quivering [35]. Marking behavior, both with urine or feces, is usually performed on items of social significance, such as places where outdoor cats are detectable, areas of the home where conflicting interactions have occurred, or on items containing the scent of other human or pets’ household members [31]. Treating cat marking behavior can be complicated and require medical therapy [32]. However, distinguishing between spraying and house-soiling elimination is not always easy [35], and from our survey findings, we are not able to distinguish between them.

Other limitations may be identified with the current study, many of which are common to survey-based surveys. The survey was disseminated through social media (Facebook, Instagram, WhatsApp, and LinkedIn), associations, and veterinary institutions using posts or emails referring to a “Questionnaire on cat behavioral problems”. This survey title may have induced a selection bias by preferentially recruiting people who are aware of their cats’ behavioral problems and perceive house-soiling as a behavioral problem. Hence, the reported findings may overestimate the prevalence of house-soiling. Moreover, house-soiling and health problems were identified by participant recall; hence, the diagnosis and prevalence of problems may not be accurate, and the technique is vulnerable to recall bias. Some Italian regions, especially the Southern ones, were less represented in our responses; however, this could reflect greater outdoor cat management (and thus less use of litter boxes) in the Southern regions than the Northern ones, which are more urbanized. The self-categorization of respondents as “professionals” or “amateurs” may not be accurate, with some breeders owning only a few cats who may have considered themselves amateurs. Moreover, because there is no official estimate of the prevalence of house-soiling in cats other than in cats presented to veterinary clinics to treat behavioral problems, the calculation of a representative sample size for our survey could not be precise. Finally, as mentioned above, some important questions like the possibility to go outdoors, the distance between the litter box and the feeding points, and the depth of the litter were not included. Designing the ideal survey is hard since a survey that is too long tends to not be completed, but these questions should be added in the future. Surveys often need to be piloted in a study to be improved in their design for future research. Notwithstanding these limitations, our study has generated important insight into pet cat demographic features, owners’ litter management details, and the prevalence of house-soiling in a representative sample of 3106 Italian pet cats.

## 5. Conclusions

This survey described the demographic characteristics, the living environment, the owners’ litter management, and the prevalence of house-soiling in a representative population of Italian domestic cats, comparing amateurs and professionals. Italian cat owners appeared to be owning multiple cats or cats and dogs simultaneously within their household. Most cats were European breeds and adults, and covered litter boxes filled with clumping substrates were most often used. Litter box cleaning management reported by our respondents was in line with what has been reported in the scientific literature as optimal, although more caution should be applied, especially in cases of multi-cat households. The differences in cat characteristics and management found between amateurs and professionals were expected and in line with the financial or non-financial purpose of their cat ownership. In general, the prevalence of inappropriate elimination was found to be lower than in other studies, suggesting that Italian cat owners and breeders manage their litter in the majority of cases as suggested in the literature. However, further studies are needed to understand the factors that may increase or decrease the risk of house-soiling.

## Figures and Tables

**Table 1:** Breakdown of all respondents’ answers. Percentages refer to a total of 3106 cats unless otherwise specified (i.e., missing values).

Variable Name	Category	Count	Percentage
		(3106 Responses)	
Origin	North	1547	52.74%
	Center	969	33.04%
	South	263	8.97%
	Other	154	5.25%
	Number of answers	2933	100%
	Missing values	173	5.57%
Described cat per respondent	1	2526	81.33%
	2	448	14.42%
	3	132	4.25%
	Number of answers	3106	100%
Housing type	Apartment	2167	69.86%
	Single family house	698	22.50%
	Multiple family house	237	7.64%
	Number of answers	3102	100%
	Missing values	4	0.13%
Housing size	≤70	685	22.53%
	71–100	1097	36.07%
	101–150	732	24.07%
	≥151	527	17.33%
	Number of answers	3041	100%
	Missing values	65	2.09%
Garden	Yes	947	30.49%
	No	2159	69.51%
	Number of answers	3106	100%
Number of adults	1	401	13.04%
	2	1466	47.66%
	3	674	21.91%
	4 or more	535	17.39%
	Number of answers	3076	100%
	Missing values	30	0.97%
Children under 7 years old	Presence	253	8.15%
	Absence	2853	91.85%
	Number of answers	3106	100%
Children between 7 and 12 years old	Presence	303	9.76%
	Absence	2803	90.24%
	Number of answers	3106	100%
Other animals	Yes	2295	73.89%
	No	811	26.11%
	Number of answers	3106	100%
Animals other than cats	Yes	938	30.20%
	No	2168	69.80%
	Number of answers	3106	100%
Dogs	Yes	689	23.07%
	No	2297	76.93%
	Number of answers	2986	100%
	Missing values	120	3.86%
Number of dogs	0	2292	76.76%
	1	439	14.70%
	2	159	5.32%
	3 or more	96	3.22%
	Number of answers	2986	100%
	Missing values	120	3.86%
Animals other than cats and dogs	Yes	208	6.97%
	No	2778	93.03%
	Number of answers	2986	100%
	Missing values	120	3.86%
Relationship with the cat	Amateur	2853	91.85%
	Professional	253	8.15%
	Number of answers	3106	100%
Number of cats	Cat living alone	995	32.03%
	Small group	1393	44.85%
	Large group	718	23.12%
	Number of answers	3106	100%
Square meters per cat	<25	547	17.99%
	25–49	979	32.19%
	50–99	963	31.67%
	≥100	552	18.15%
	Number of answers	3041	100%
	Missing values	65	2.09%
Cat’s gender	Female	287	9.26%
	Neutered female	1366	44.08%
	Male	208	6.71%
	Neutered male	1238	39.95%
	Number of answers	3099	100%
	Missing values	7	0.23%
Cat’s breed	European	1573	50.64%
	Maine Coon	175	5.63%
	Mixed-breed	593	19.09%
	Persian	110	3.54%
	Siberian	114	3.67%
	Other	541	17.42%
	Number of answers	3106	100%
Length of cat’s hair	Short	2471	79.89%
	Long	622	20.11%
	Number of answers	3093	100%
	Missing values	13	0.42%
Cat’s age	<2 years	785	25.70%
	2–5 years	981	32.12%
	>5 years	1288	42.17%
	Number of answers	3054	100%
	Missing values	52	1.67%
Number of litter boxes	1	1470	47.33%
	2	817	26.30%
	3	388	12.49%
	4 or more	431	13.88%
	Number of answers	3106	100%
Type of litter box	Open	1223	39.38%
	Covered	1596	51.38%
	Open and Covered	276	8.89%
	Other	11	0.35%
	Number of answers	3106	100%
Box location	Balcony	600	19.48%
	Bathroom	1599	51.92%
	Kitchen	234	7.60%
	Basement	106	3.44%
	Living room	424	13.77%
	Bedroom	951	30.88%
	Entrance	224	7.27%
	Stairs	47	1.53%
	Garden	47	1.53%
	Number of answers	3080	100%
	Missing values	26	0.84%
Type of litter	Clumping	1630	52.48%
	Non-clumping	420	13.52%
	Biodegradable	626	20.15%
	Silica gel	301	9.69%
	Other	129	4.15%
	Number of answers	3106	100%
Litter scooping frequency	More than twice a day	225	7.24%
	Twice a day	1069	34.42%
	Once a day	1209	38.92%
	Twice a week	241	7.76%
	Thrice a week	175	5.63%
	Other	187	6.02%
	Number of answers	3106	100%
Litter full replacement frequency	More than two/three times a week	197	6.34%
	Once a week	1639	52.77%
	Every ten/twenty days	239	7.69%
	Once a month	886	28.53%
	Other	145	4.67%
	Number of answers	3106	100%
Eliminates outside the litter	Yes	520	16.74%
	No	2586	83.26%
	Number of answers	3106	100%

**Table 2:** Breakdown of answers by respondents whose cat(s) eliminates outside the litter box. Percentages refer to a total of 520 cats eliminating outside the litter box, unless otherwise specified (i.e., missing values).

Variable Name	Category	Count	Percentage
		(520 Responses)	
Type of elimination	Urinary house-soiling	261	54.60%
	Fecal house-soiling	119	24.90%
	Concurrent expression of urinary and fecal house-soiling	98	20.50%
	Number of answers	478	100%
	Missing values	42	8.08%
Spots of the elimination when outside the litter box	Same spot	309	64.64%
	Different spots	169	35.36%
	Number of answers	478	100%
	Missing values	42	8.08%
Precise location of the elimination when outside the litter box	Bedroom	76	17.31%
	Floor	40	9.11%
	Near the litter	124	28.25%
	Objects	139	31.66%
	Absorbent mat	60	13.67%
	Number of answers	439	100%
	Missing values	81	15.58%
Posture when eliminating	Squatting	166	35.24%
	Standing with tail raised	144	30.57%
	Have not observed	161	34.18%
	Number of answers	471	100%
	Missing values	49	9.42%
Presence of cat’s health problem	Yes	89	17.12%
	No	360	69.23%
	I do not know	71	13.65%
	Number of answers	520	100%
Type of cat’s health problem	Healthy	360	80.18%
	Urinary tract disease	40	8.91%
	Others	49	10.91%
	Number of answers	449	100%
	Missing values (I do not know)	71	13.65%

## Data Availability

The data presented in this study are available on request from the corresponding author.

## Supplementary Materials

The following supporting information can be downloaded at https://www.mdpi.com/article/10.3390/ani13142382/s1, Table S1: Online survey of cat owners in Italy, consisting of 18 closed and 3 open-ended questions. Table S2: Distribution and promotion of the survey. The survey was disseminated by means of Italian invitation letters and links through social media, animal associations, and veterinary institutions. Table S3: Description and categories of the different variables considered in the descriptive analysis. Table S4: Frequency table of the explanatory variable (i.e., litter scooping frequency) related to the number of cats. Table S5: Frequency table of the explanatory variables related to the relationship with the cat (i.e., amateur, professional).
