# Supplementary material for: Litter Management Practices and House-Soiling in Italian Cats
Source: Animals (Basel). 2023 Jul 22;13(14):2382. doi: 10.3390/ani13142382 (PMC10376366; doi:10.3390/ani13142382)
Supplement: Supplementary file 1 [file animals-13-02382-s001.zip › animals-2486827-supplementary.pdf]

Supplementary materials

Table S1. Online survey of cat owners in Italy, consisting of 18 closed and 3 open-ended questions.

Default question block

1. Where do you live?
   - ○ Apartment (Please, specify the size __________ square meters)
   - ○ Single family house (Please, specify the size __________ square meters)
   - ○ Multiple family house (Please, specify the size __________ square meters)
   - ○ Other (Please, specify) ______________________

2. How many adults live with you? (Please, write the number, e.g., 3) __________

3. How many children under 7 years live with you? (Please, write the number, e.g., 3) __________

4. How many children between 8 and 12 years live with you? (Please, write the number, e.g., 3) __________

5. Are there other pets in the family?
   - ○ Yes (Please, specify species and number of other animals) ______________________
   - ○ No

6. What is your relationship with the cat/s?
   - ○ Professional (e.g., breeder)
   - ○ Amateur (e.g., owner)

7. How many cats do you own?
   - ○ 0
   - ○ 1
   - ○ 2
   - ○ 3
   - ○ Other (Please, specify as number) ______________________

Cat details (if you have more than one cat, please, answer the following questions referring to only one cat)

8. What gender is your cat?
   - ○ Female
   - ○ Neutered female
   - ○ Male
   - ○ Neutered male

9. What breed is your cat?
   - ○ Persian Cat Breed
   - ○ Chartreux Cat Breed (Carthusian)
   - ○ Siamese Cat Breed
   - ○ Mixed Cat Breed
   - ○ European Cat Breed
   - ○ Other (Please, specify the breed) ____________________

10. How old is your cat?
    - ○ Younger than 6 months
    - ○ Between 6 months and 2 years
    - ○ Between 2 and 5 years
    - ○ Older than 5 years

11. How many litter boxes do you have in your house?
    - ○ 1
    - ○ 2
    - ○ 3
    - ○ Other__________ (Please, specify as number)

12. What type of litter box does your cat use?
    - ○ Open
    - ○ Covered

- ○ Other (Please, specify) ____________________

13. Where is the litter box(s) located? (Multiple answers allowed)
    - □ Balcony
    - □ Bathroom
    - □ Kitchen
    - □ Basement
    - □ Living room
    - □ Other (Please, specify) ____________________

14. What type/brand of cat litter substrate do you use?
    - ○ Clumping
    - ○ Non-clumping
    - ○ With minerals
    - ○ Plant-based
    - ○ Silica gel
    - ○ Other (Please, specify) ____________________

15. How often do you scoop the litter?
    - ○ Once a day
    - ○ Twice a day
    - ○ Twice a week
    - ○ Thrice a week
    - ○ Other (Please, specify) ____________________

16. How often do you wash the box and replace the litter?
    - ○ Once a day
    - ○ Once a week
    - ○ Once a month
    - ○ Other (Please, specify) ____________________

17. Does your cat eliminate outside the litter box?
    - ○ Yes
    - ○ No

18. (If you answered "Yes" to the previous question) What does the cat eliminate outside the litter box?
    - ○ Urine
    - ○ Faeces
    - ○ Both

19. Where does the cat eliminate?
    - ○ Same spot (Please, specify) ____________________
    - ○ Different spots (Please, specify) ____________________

20. What posture does the cat take when eliminating outside the litter box?
    - ○ Squatting
    - ○ Standing with tail raised
    - ○ I have not observed
    - ○ Other (Please, specify) ____________________

21. Does the cat have some health problems?
    - ○ Yes (Please, specify the health problem/s) ____________________
    - ○ No
    - ○ I do not know

Table S2. Distribution and promotion of the survey. The survey was disseminated by means of Italian invitation letters and links through social media, animal associations, and Veterinary Institutions.

Category	Name	Method
Entertainment Facebook pages	Gatto: l'animale perfetto. Quanti gattari siamo?<br>Gatti<br>Gatti abbandonati e trovati in Romagna<br>I Gatti Conquisteranno il Mondo (L'esercito!)<br>Canini&Gattini<br>Amore di Gatti.... mi@o mi@o!!!<br>Gattivissimi noi! Gruppo di svago per chi è stregatto dai gatti<br>Micimiao Bologna - i gatti della tua città<br>Gatti in sicurezza! Chi li ama, li protegge! Il Gruppo Originale!<br>Regalo o cerco gatti in adozione<br>Mamme vet<br>Hai un gatto se...<br>Amici dei gatti<br>Gatti con personalità<br>Adotta in Romagna<br>Amici per la vita<br>Sphynx & Maine Coon in italia felini speciali	Posting the invitation letter and link on the group page
Vet Facebook pages	Veterinari Pugliesi<br>Bestiario Veterinario<br>Cogito Ergo Vet	Posting the invitation letter and link on the group page
Personal Facebook pages	Authors personal pages	Posting the invitation letter and link on the personal page
Entertainment Instagram profiles	Gattini&Canetti_cerebrolesetti<br>G come gatto<br>Gatto con personalità<br>Miciamici_bologna_odv	Publishing the invitation letter and the link through Instagram Stories (24 h), Instagram Stories Highlights or Posts
Cat Associations Instagram profiles	Pagina del gattile di Budrio Castenaso<br>Gattile oasi felina Pesaro	Publishing the invitation letter and the link through Instagram Stories (24 h), Instagram Stories Highlights or Posts
Cat Breeders Instagram profiles	Allevamento gatti siberiani<br>Chez Blanchette	Publishing the invitation letter and the link through Instagram Stories (24 h), Instagram Stories Highlights or Posts
Personal Instagram profiles	Authors personal pages<br>Eleonora Montagnana personal page	Publishing the invitation letter and the link through Instagram Stories (24 h), Instagram Stories Highlights or Posts
Personal LinkedIn profile	Author personal page	Posting the invitation letter and link on the personal page
Personal WhatsApp contacts	Author contacts (groups/individuals)	Spreading the invitation letter and link via message
Cat Associations	Associazioni Feline Federate (AFeF)<br>Associazione Feline Italiana	Posting the invitation letter and link on their website and FB page

		Emailing the invitation letter with the link to their members and website
Veterinary institution	Ordine dei Medici Veterinari della Provincia di Pisa	Emailing the invitation letter and the link to their members

Table S3. Description and categories of the different variables considered in the descriptive analysis.

Variable name	Description	Categories
**Respondents' details**		
Origin	The geographic region of Italy from which the respondent replied to the survey.	North, Center, South, Other*
Described cat per respondent	Number of times the respondent filled in the questionnaire (for one or more than one cat).	1, 2, 3
**Respondents' housing details**		
Housing type	Type of the respondent's housing.	Apartment, Single family house, Multiple family house
Housing size	Size of the respondent's housing in square meters.	≤70, 71-100, 101-150, ≥151
Garden	Does the respondent have a garden?	Yes/No
**Respondents' family details**		
Number of adults	How many adults live in the housing?	1, 2, 3, 4 or more
Children under 7 years old	Are there children under the age of 7 years?	Presence/Absence
Children between 7 and 12 years old	Are there children between the age of 7 and 12 years?	Presence/Absence
**Respondents' pets details**		
Other animals	Are there other pets in the family?	Yes/No
Animals, other than cats	Are there pets other than cats in the family?	Yes/No
Dogs	Is there a dog in the family?	Yes/No
Number of dogs	What is the number of dogs in the family?	0, 1, 2, 3 or more
Animals, other than cats and dogs	Are there animals other than cats and dogs?	Yes/No
**Cat's respondent details**		
Relationship with the cat	What is the relationship of the respondent with the cat?	Amateur, Professional
Number of cats	How many cats does the respondent own?	Cat living alone (1), Small group (2-3), Large group (4+)
Square meters per cat	The surface of the respondent's housing, in m2, divided by the number of cats owned.	<25, 25-49, 50-99, ≥100

Cat's gender	What is the gender of the cat?	Female, Neutered Female, Male, Neutered Male
Cat's breed	What is the breed of the cat?	European, Maine Coon, Mixed-breed, Persian, Siberian, Other
Length of cat's hair	Cat's hair length, defined by the cat breed.	Short, Long
Cat's age	How old is the cat?	<2 years, 2-5 years, >5 years
Presence of cat's health problems	Does the cat have any health problems?	Yes/No/I do not know
Type of cat's health problem	If yes, what health problem?	Healthy, Urinary tract disease, Others
	**Litter details**	
Number of litter boxes	How many litter boxes does the respondent have?	0, 1, 2, 3, 4 or more
Type of litter box	What is the type of litter box(es)?	Open, Covered, Open and Covered, Other
Litter box location	Where is(are) the litter box(es) located?	Yes/No to all locations listed
	Balcony, bathroom, kitchen, basement, living room, bedroom, entrance, stairs, garden	
Type of litter	What type/brand of cat litter substrate does the respondent use?	Clumping, Non-clumping, Biodegradable, Silica gel, Other
Litter scooping frequency	How often does the respondent scoop the litter?	More than twice a day, Twice a day, Once a day, Twice a week, Thrice a week, Other
Litter full replacement frequency	How often does the respondent wash the box and replace the litter?	Less than two/three times a week, Once a week, Every ten/twenty days, Once a month, Other
Eliminates outside the litter box	Does the cat eliminate outside the litter box?	Yes, No
Type of elimination	If the cat eliminates outside the litter box, what type of elimination is performed?	Urinary house-soiling, Faecal house-soiling, Concurrent expression of urinary and faecal house-soiling
Spots of the elimination when outside the litter box	Where does the cat eliminate?	Same spot, Different spots
Precise location of the elimination when outside the litter box	Where does the cat eliminate precisely?	Bedroom, Floor, Near the litter, Objects, Outside the house, Absorbent mat
Posture when eliminating	Posture when the cat eliminates outside the litter box?	Squatting, Standing with tail raised, Have not observed

Notes: Other* = Italian islands and Italians living abroad.

Table S4. Frequency table of the explanatory variable "litter scooping frequency" related to the number of cats.

Explanatory Variable	Cat living alone	Small group	Large group
Litter scooping frequency*** (X^2 = 176.25)	More than twice a day 31/3106 (3.1%)<br>Twice a day 283/3106 (28.5%)<br>Once a day 444/3106 (44.6%)<br>Twice a week 94/3106 (9.5%)<br>Thrice a week 67/3106 (6.7%)<br>Other 76/3106 (7.6%)	More than twice a day 81/3106 (5.8%)<br>Twice a day 483/3106 (34.7%)<br>Once a day 548/3106 (39.3%)<br>Twice a week 115/3106 (8.3%)<br>Thrice a week 81/3106 (5.8%)<br>Other 85/3106 (6.1%)	More than twice a day 113/3106 (15.7%)<br>Twice a day 303/3106 (42.2%)<br>Once a day 217/3106 (30.2%)<br>Twice a week 32/3106 (4.5%)<br>Thrice a week 27/3106 (3.8%)<br>Other 26/3106 (3.6%)

Legend: * = p < 0.05, ** = p < 0.01, *** = p < 0.001

Table S5. Frequency table of the explanatory variables related to the relationship with the cat (i.e., amateur, professional).

Explanatory Variables	Amateur	Professional
Origin* (X^2 = 7.89)	North 1419/2705 (52.5%)<br>Center 889/2705 (32.9%)<br>South 254/2705 (9.4%)<br>Other 143/2705 (5.3%)	North 128/228 (56.1%)<br>Center 80/228 (35.1%)<br>South 9/228 (3.9%)<br>Other 11/228 (4.8%)
Described cat per respondent (X^2 = 0.17)	1 – 2321/2853 (81.2%)<br>2 – 412/2853 (14.4%)<br>3 – 120/2853 (4.2%)	1 – 205/253 (81.0%)<br>2 – 36/253 (14.2%)<br>3 – 12/253 (4.7%)
Housing type*** (X^2 = 36.42)	Apartment 2030/2849 (71.3%)<br>Single family house 604/2849 (21.2%)<br>Multiple family house 215/2849 (7.5%)	Apartment 137/253 (54.2%)<br>Single family house 94/253 (37.2%)<br>Multiple family house 22/253 (8.7%)
Housing size*** (X^2 = 107.63)	≤70 – 672/2790 (24.1%)<br>71-100 - 1023/2790 (36.7%)<br>101-150 - 663/2790 (23.8%)<br>≥151 - 432/2790 (15.5%)	≤70 – 13/251 (5.2%)<br>71-100 - 74/251 (29.5%)<br>101-150 - 69/251 (27.5%)<br>≥151 - 95/251 (37.8%)
Garden*** (X^2 = 30.66)	Yes 831/2853 (29.1%)<br>No 2022/2853 (70.9%)	Yes 116/253 (45.8%)<br>No 137/253 (54.2%)
Number of adults*** (X^2 = 43.39)	1 – 386/2824 (13.7%)<br>2 - 1355/2824 (48.0%)<br>3 - 627/2824 (22.2%)<br>4 or more - 456/2824 (16.1%)	1 – 15/252 (6.0%)<br>2 - 111/252 (44.0%)<br>3 - 47/252 (18.7%)<br>4 or more - 79/252 (31.3%)
Children under 7 years old (X^2 = 0.29)	Presence 228/2853 (8.0%)<br>Absence 2625/2853 (92.0%)	Presence 25/253 (9.9%)<br>Absence 228/253 (90.1%)
Children between 7 and 12 years old*** (X^2 = 22.22)	Presence 257/2853 (9.0%)<br>Absence 2596/2853 (90.1%)	Presence 46/253 (18.2%)<br>Absence 207/253 (81.8%)
Other animals*** (X^2 = 85.90)	Yes 2046/2853 (71.7%)<br>No 807/2853 (28.3%)	Yes 249/253 (98.4%)<br>No 4/253 (1.6%)
Animals, other than cats*** (X^2 = 28.85)	Yes 824/2853 (28.9%)<br>No 2029/2853 (71.1%)	Yes 114/253 (45.1%)<br>No 139/253 (54.9%)
Dogs*** (X^2 = 42.12)	Yes 593/2746 (21.6%)<br>No 2153/2746 (78.4%)	Yes 96/240 (40.0%)<br>No 144/240 (60.0%)
Number of dogs*** (X^2 = 42.17)	0 – 2148/2746 (78.2%)<br>1 – 380/2746 (13.8%)<br>2 – 134/2746 (4.9%)<br>3 or more – 84/2746 (3.1%)	0 – 144/240 (60.0%)<br>1 – 59/240 (24.6%)<br>2 – 25/240 (10.4%)<br>3 or more – 12/240 (5%)

Animals, other than cats and dogs (X^2 = 0.01)	Yes 191/2746 (7.0%)<br>No 2555/2746 (93.0%)	Yes 17/240 (7.1%)<br>No 223/240 (92.9%)
Number of cats*** (X^2 = 575.16)	Cat living alone 992/2853 (34.8%)<br>Small group 1355/2853 (47.5%)<br>Large group 506/2853 (17.7%)	Cat living alone 3/253 (1.2%)<br>Small group 38/253 (15.0%)<br>Large group 212/253 (83.8%)
Square meter per cat*** (X^2 = 364.06)	<25—395/2790 (14.2%)<br>25-49—906/2790 (32.5%)<br>50-99—939/2790 (33.7%)<br>≥100—550/2790 (19.7%)	<25—152/251 (60.6%)<br>25-49—73/251 (29.1%)<br>50-99—24/251 (9.6%)<br>≥100—2/251 (0.8%)
Cat's gender*** (X^2 = 981.16)	Female 138/2847 (4.8%)<br>Neutered female 1342/2847 (47.1%)<br>Male 151/2847 (5.3%)<br>Neutered male 1216/2847 (42.7%)	Female 149/252 (59.1%)<br>Neutered female 24/252 (9.5%)<br>Male 57/252 (22.6%)<br>Neutered male 22/252 (8.7%)
Cat's breed*** (X^2 = 657.83)	European 1570/2853 (55.0%)<br>Maine Coon 106/2853 (3.7%)<br>Mixed-breed 591/2853 (20.7%)<br>Persian 76/2853 (2.7%)<br>Siberian 89/2853 (3.1%)<br>Other 421/2853 (14.8%)	European 3/253 (1.2%)<br>Maine Coon 69/253 (27.3%)<br>Mixed-breed 2/253 (0.8%)<br>Persian 34/253 (13.4%)<br>Siberian 25/253 (9.9%)<br>Other 120/253 (47.4%)
Length of cat's hair*** (X-squared = 499.74)	Short 2406/2841 (84.7%)<br>Long 435/2841 (15.3%)	Short 65/252 (25.8%)<br>Long 187/252 (74.2%)
Cat's age*** (X^2 = 18.77)	<2 years 719/2809 (25.6%)<br>2-5 years 876/2809 (31.2%)<br>>5 years 1214/2809 (43.2%)	<2 years 66/245 (26.9%)<br>2-5 years 105/245 (42.3%)<br>>5 years 74/245 (30.2%)
Number of litter boxes*** (X^2 = 872.73)	1—1455/2853 (51.0%)<br>2—799/2853 (28.0%)<br>3—357/2853 (12.5%)<br>4 or more—242/2853 (8.5%)	1—15/253 (6.0%)<br>2—18/253 (7.1%)<br>3—31/253 (12.3%)<br>4 or more—189/253 (74.7%)
Type of litter box*** (X^2 = 71.32)	Open 1152/2853 (40.4%)<br>Covered 1472/2853 (51.6%)<br>Open and Covered 218/2853 (7.7%)<br>Other 11/2853 (0.4%)	Open 71/253 (28.1%)<br>Covered 124/253 (49.0%)<br>Open and Covered 58/253 (22.9%)<br>Other 0/253 (0.0%)
Box location – Balcony*** (X^2 = 66.28)	Yes 503/2832 (17.8%)<br>No 2329/2832 (82.2%)	Yes 97/248 (39.1%)<br>No 151/248 (60.9%)
Box location – Bathroom*** (X^2 = 11.20)	Yes 1445/2832 (51.0%)<br>No 1387/2832 (49.0%)	Yes 154/248 (62.1%)<br>No 94/248 (37.9%)
Box location – Kitchen*** (X^2 = 77.22)	Yes 180/2832 (6.4%)<br>No 2652/2832 (93.6%)	Yes 54/248 (21.8%)<br>No 194/248 (78.2%)
Box location – Basement*** (X^2 = 44.99)	Yes 79/2832 (2.8%)<br>No 2753/2832 (97.2%)	Yes 27/248 (10.9%)<br>No 221/248 (89.1%)
Box location – Living room*** (X^2 = 141.36)	Yes 328/2832 (11.6%)<br>No 2504/2832 (88.4%)	Yes 96/248 (38.7%)<br>No 152/248 (61.3%)
Box location – Bedroom*** (X^2 = 116.89)	Yes 799/2832 (28.2%)<br>No 2033/2832 (71.8%)	Yes 152/248 (61.3%)<br>No 96/248 (38.7%)
Box location – Entrance*** (X^2 = 23.39)	Yes 187/2832 (6.6%)<br>No 2645/2832 (93.4%)	Yes 37/248 (14.9%)<br>No 211/248 (85.1%)
Box location – Stairs (X^2 = 0.18)	Yes 44/2832 (1.6%)<br>No 2788/2832 (98.4%)	Yes 3/248 (1.2%)<br>No 245/248 (98.8%)
Box location – Garden (X^2 = 0.93)	Yes 45/2832 (1.6%)<br>No 2787/2832 (98.4%)	Yes 2/248 (0.8%)<br>No 246/248 (99.2%)
Type of litter***	Clumping 1493/2853 (52.3%)	Clumping 137/253 (54.2%)

(X^2 = 22.10)	Non-clumping 395/2853 (13.8%)<br>Biodegradable 561/2853 (19.7%)<br>Silica gel 292/2853 (10.2%)<br>Other 112/2853 (3.9%)	Non-clumping 25/253 (9.9%)<br>Biodegradable 65/253 (25.7%)<br>Silica gel 9/253 (3.6%)<br>Other 17/253 (6.7%)
Litter scooping frequency***<br>(X^2 = 101.70)	More than twice a day 186/2853 (6.5%)<br>Twice a day 932/2853 (32.7%)<br>Once a day 1144/2853 (40.1%)<br>Twice a week 241/2853 (8.4%)<br>Thrice a week 170/2853 (6.0%)<br>Other 180/2853 (6.3%)	More than twice a day 39/253 (15.4%)<br>Twice a day 137/253 (54.2%)<br>Once a day 65/253 (25.7%)<br>Twice a week 0/253 (0.0%)<br>Thrice a week 5/253 (2.0%)<br>Other 7/253 (2.8%)
Litter full replacement frequency***<br>(X^2 = 29.42)	More than two/three times a week 171/2853 (6.0%)<br>Once a week 1480/2853 (51.9%)<br>Every ten/twenty days 223/2853 (7.8%)<br>Once a month 847/2853 (29.7%)<br>Other 132/2853 (4.6%)	More than two/three times a week 26/253 (10.3%)<br>Once a week 159/253 (62.8%)<br>Every ten/twenty days 16/253 (6.3%)<br>Once a month 39/253 (15.4%)<br>Other 13/253 (5.1%)
Eliminates outside the litter box<br>(X^2 = 0.06)	Yes 479/2853 (16.8%)<br>No 2374/2853 (83.2%)	Yes 41/253 (16.2%)<br>No 212/253 (83.8%)

Legend: * = $p < 0.05$, ** = $p < 0.01$, *** = $p < 0.001$
